# Progress in Medicine

**Published:** 1899-01-28

**Authors:** 


### Progress in Medicine.

### RENAL MEDICINE.

(*Continued from page* 278.)

**Treatment.**—Ewart,[15] in the case of chronic tubal nephritis, aims at drawing the œdema into the lower limbs and affording it a free exit by deep incisions. This gives physiological rest to the kidney by carrying off effete matters through the lymphatics. Meanwhile, the general health and heart action are supported, and where uræmia without œdema occurs he would advise either venesection or artificial œdema and drainage. Strict antiseptic precautions are used in all cases, and the dependent posture of the limbs enforced by raising the head of the bed six or eight inches.[16] He gives at first only milk, but when a free flow is started he allows a generous and varied diet with wine, active exercise, and massage, so long as the free drainage is kept up. In the Edinburgh discussion Tirard[17] advised the use of woollen clothing and waist belts in order to prevent the acute attacks to which chronic tubal nephritis is liable. Over-exertion and mental depression must be guarded against, and the appetite encouraged rather than irritated by too strict a dietary. So little success has attended any treatment based on the pathology of the disease that he only advises the ordinary diuretics and purgatives. For headache nitro-glycerine is most useful, but it has little power in cutting short uræmic attacks, for which a rapidly active purgative is our most efficient remedy. On the whole, he is opposed to the use of morphia on theoretical grounds. Ewald, of Berlin, advocated repeated tapping and venesection, especially where uræmia is present. By means of large drainage tubes under antiseptic precautions he provides a permanent outflow from the œdematous limbs, tapping the pleura frequently if any accumulation take place, and finds this method highly successful, even producing permanent cures; while for uræmia, if the condition of the patient allows and other plans fail, he advocates venesection combined with saline injections. T. Barr relies upon a carbohydrate diet with salol, and never gives pilocarpine from its dangerous results. He would limit the employment of venesection to some cases of high tension. McVail prefers plain water as a diuretic, and no longer uses a prolonged milk diet. Both opium and pilocarpin have given him good results in uræmia. C. T. Macalister recommends oxygen inhalations, and never allows pilocarpin if the patient is unconscious, or morphia if the pupils are contracted. He notes that in uræmic urine the specific gravity is no guide to the urea passed; the higher it is the less urea is contained in it, while T. M. Allison finds that some cases benefit by the moderate use of thyroid extract. Dreschfeld,[18] in a paper on diuretin, claims good results from it in mitral disease, recent ascites from cirrhosis, and in acute and parenchymatous Bright's disease. In scarlatinal and interstitial nephritis it is of little use. He employs 10 or 12 grain doses three times a day, carefully increased even to 30 grain doses.

W. McEnroe[19] speaks highly of the effects of perchloride of mercury in cirrhosis of the kidney. Given with the perchloride of iron he claims that the pulse becomes soft, the specific gravity of the urine rises rapidly, and the patient makes steady improvement. For the headache he advises ergot. Two remarkable cases of uræmia are discussed by A. T. Wilkinson,[20] as showing the possibility of a good recovery in some cases, and the absence of organic disease in others. He believes that attacks may occur from senile kidney rather than from a really diseased one; that without any sign of kidney disease except the high tension serious uræmia may develop, and that the prognosis in such cases is fairly good. It seems uncertain whether the cause of such attacks lies in excessive formation of the uræmic poison rather than in failure of elimination. He does not believe in the uric acid theory, but attacks of this kind do occur without true kidney disease, and he would recommend for them calomel, salines, bleeding, and a careful diet. An acute case of uræmia following after an attack of dysentery, and treated by saline injections, is reported by Potienko.[21] The urine, which had been normal previous to the acute symptoms, showed albumen casts and blood. Improvement followed every injection, and a complete recovery was obtained, the albumen disappearing, and the patient regaining his ordinary condition.

**Urology.**—G. F. Laidlaw[22] thinks that more attention should be paid to the elimination of phosphates in Bright's disease than to the fall in the urea. He points out that it is the most constant symptom in interstitial nephritis, where indeed albumen and a low specific gravity may or may not be found. He adds that no case of interstitial nephritis has ever been found in which the excretion of phosphates was normal. Both in this disease and in waxy kidney the phosphates are always reduced. Unfortunately there are other diseases in which the symptom occurs. Such are the acute fevers, pregnancy, cirrhosis of the liver, gout, and lead poisoning. The estimation of the salt can be easily made by the uranium nitrate test. Buckner[23] gives a simple means for estimating the amount of urea. If the other salts in the urine, especially the chlorides, are in normal quantities, urea forms one half of the total, unless some abnormal constituent is present, such as sugar. As the specific gravity gives the total percentage of solids, we merely test for the chlorides with nitrate of silver, and allow for their excess or defect, and for any abnormal matter present, and then take half the remainder of the solids to be urea. Thus he calculates for normal urine, where the sp. gr. =1,028, the chlorides being normal, and a thousand c.c. are passed in 24 hours, that the day's solids are 28 × 2·33 = 6·64 per cent., and the urea is 3·3 grammes per cent. The writer, from numerous experiments, believes that similar calculations will give practically correct results. J. B. Nicholls[24] draws attention to the fact that carbon dioxide is a normal and constant constituent of urine, either in a free form or as a carbonate, the former only in strongly acid urine, and the latter in all other cases. The free acid may amount to from 2 to 12 per cent. by volume. The source may be from the metabolism of the body or from food, as well as from fermentation of the urea after excretion.

Indol and indican have been the subjects of several investigations of late. J. Leonard Vaux[25] finds that the former is derived, not only from the proteids of food, but also from various forms of suppuration in the body. The source of amyloid matter, as seen in the waxy kidney, must now be looked for in the action of indol, or a derivative of it, upon degenerated cells. When acted on by iodine this substance is changed to indigo, and, if sulphuric acid be added, to indigo blue. This is, of course, what is seen in the common test for amyloid degeneration. Normally the liver is able to oxydise the indol produced, but if it fails to do so a quantity of indigo-forming substances pass into the circulation, which may appear as indoxyl sulphate in the urine, or produce in time amyloid degeneration. Herter[26] agrees that the indican of the urine is derived from indol, the seat of conversion probably being the liver. Among the sources of indol in the body the action of the colon bacillus on proteid food stuffs is important. He does not regard it as a highly poisonous substance, but severe headache and depression are produced by it when given in large doses experimentally.

[15] Lancet, July 2. [16] Brit. Med. Jour., Oct. 8. [17] Ibid. [18] Lancet, June 11. [19] Med. Rec., June 25. [20] Lancet, June 4. [21] Les Nouv. Remèdes, Nov. 8. [22] Med. Rec., Sept. 3. [23] Med. Rec., p. 589, Sept. [24] Med. Rec., May 14. [25] Brit. Med. Jour., July 4. [26] New York Med. Jour., July 16.

